# In sickness and in health: pivotal role of vitamin D

**DOI:** 10.11613/BM.2020.020501

**Published:** 2020-06-15

**Authors:** Nora Nikolac Gabaj, Adriana Unic, Marijana Miler, Tomislav Pavicic, Jelena Culej, Ivan Bolanca, Davorka Herman Mahecic, Lara Milevoj Kopcinovic, Alen Vrtaric

**Affiliations:** 1 Department of Clinical Chemistry, Sestre milosrdnice University Hospital Center, Zagreb, Croatia; 2 Faculty of Pharmacy and Biochemistry, University of Zagreb, Zagreb, Croatia; 3 Department of Human Reproduction, Sestre milosrdnice University Hospital Center, Zagreb, Croatia; 4 Department for Endocrinology, Dieabetes and Metabolism, Sestre milosrdnice University Hospital Center, Zagreb, Croatia

**Keywords:** vitamin D, preanalytical variability, diabetes mellitus, polymorphism, fertility, extravascular body fluids

## Abstract

Within the last several years, frequency of vitamin D testing has multiplied substantially all over the world, since it has been shown to have an important role in many diseases and conditions. Even though liquid chromatography - tandem mass spectrometry (LC-MS/MS) has been identified as “gold standard” method for vitamin D measurement, most laboratories still use immunochemistry methods. Besides analytical problems (hydrophobicity, low circulating concentrations, ability to bind to lipids, albumins and vitamin D binding protein, presence of multiple vitamin D metabolites and variable ratios of 25(OH)D_2_ and 25(OH)D_3_ in the blood), vitamin D shows great preanalytical variability, since its concentration is drastically influenced by seasonal changes, exposure to sun, type of clothes or sun block creams. Vitamin D is mostly measured in serum or plasma, but new studies are showing importance of measuring vitamin D in pleural effusions, breast milk, urine, synovial fluid and saliva. Besides the main role in calcium homeostasis and bone metabolism, many studies linked vitamin D deficiency with cancer, cardiovascular diseases, diabetes, fertility and many other conditions. However, even though initial observational studies indicated that supplementation with vitamin D might be beneficial in disease development and progression; first results of well-designed randomized controlled prospective studies did not find differences in frequency of cardiovascular events or invasive cancer between patients taking vitamin D supplementation compared to placebo. In the light of these recent findings, validity of excessive vitamin D testing remains an open question.

## Introduction

Prolific scientific activity has always been one of the main missions of the Croatian Society of Medical Biochemistry and Laboratory Medicine (CSMBLM). For more than 60 years, this society aims to educate their members on the relevant topics in the field of laboratory medicine. One of the longest continuous courses organized under the CSMBLM auspices is the annual symposium, which has been held in Zagreb in September 2019 for the 30th time. Each year, laboratory specialists, together with clinical experts from one hospital in Croatia are given the opportunity to present their knowledge on a specific topic from the fields of clinical chemistry, haematology, coagulation, immunology, toxicology or molecular diagnostics. The topic of the last symposium, organized by the Sestre milosrdnice University Hospital Center from Zagreb, was vitamin D. This paper gives an overview of the lectures presented at the symposium.

## Vitamin D yesterday, today, tomorrow

In the last decade, frequency, as well as the budget for vitamin D testing increased significantly worldwide (*1*). The explosion of vitamin D utilization is a result of many promising observational studies that have associated vitamin D concentration with health benefits in cardiovascular diseases, cancer, diabetes, fertility, and many others (*2*). The number of published papers on vitamin D, as well as diseases associated with its deficiency, increases daily (*3*).

In 2016, several professional medical societies issued Croatian national guidelines for prevention, detection and therapy of vitamin D deficiency in adults (*4*). Based on the cut-off values from the guidelines, vitamin D concentrations lower than 30 nmol/L are considered as an extreme deficiency, < 50 nmol/L as deficiency and < 75 nmol/L as insufficiency. One should be aware that these cut-off values are not obtained on the evidence-based principle and most societies and organizations refer to the single point of origin when defining recommended vitamin D concentrations, resulting in a high number of vitamin D deficient individuals (*2*). Recommended daily intake of vitamin D for adults is 600 IU, and if sufficient amount is not ingested by food, supplements should be taken (*4*).

The usage of dietary supplements has become more increasing in recent years (*5*). A large European multicenter study published in 2018 on patient’s knowledge and awareness about the effect of the over-the-counter drugs and dietary supplements on laboratory test results, has revealed some alarming issues (*6*). More than two thirds of patients are taking at least one dietary supplement, but they are usually not reporting the usage neither to their physicians nor to the laboratory staff. Our recent investigation has shown that immunochemistry methods for vitamin D measurement might react differently in patients taking vitamin D supplements and in patients without therapy (*7*). Therefore, usage of any dietary supplements should be reported to laboratory staff.

Recently however, clinical usefulness of vitamin D supplementation therapy has been questioned, since first results of well-designed randomized controlled prospective studies have been published. Surprisingly, one of the largest studies (N = 25,871) did not find differences in the frequency of cardiovascular events or invasive cancer between patients taking vitamin D supplementation compared to placebo (*8*). These findings might have long-term effects on the future of vitamin D testing.

## Vitamin D metabolism and mechanism of action

**Figure 1:**
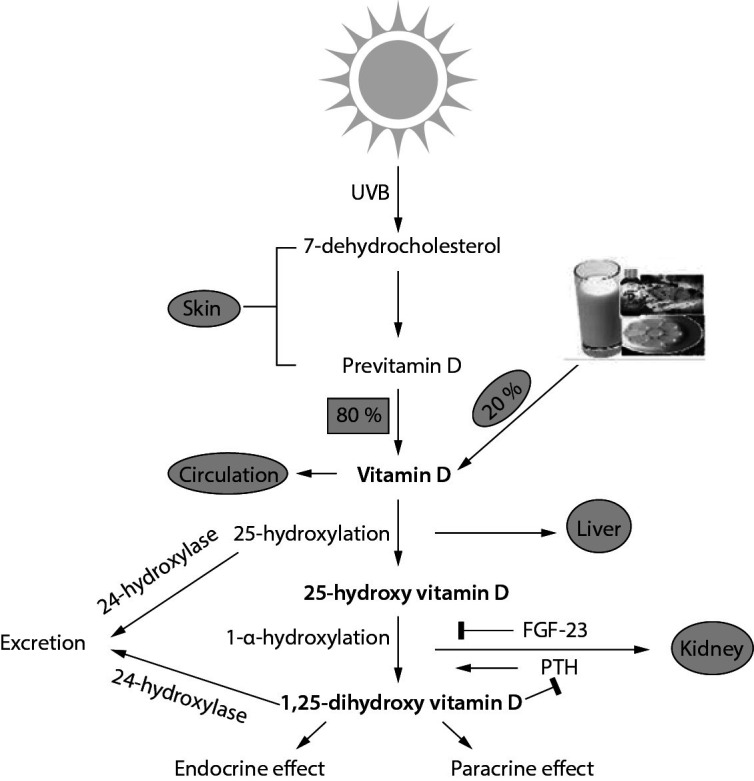
Vitamin D metabolism. 80% of vitamin D is produced in the skin in response to ultraviolet B exposure and 20% of vitamin D is ingested by food or supplementation. In circulation vitamin D binds to the vitamin D-binding protein and is transported to the liver, where is hydroxylated to 25-hydroxy vitamin D and then to the kidney where 1,25-dihydroxy vitamin D is formed. 1-α hydroxylation process is tightly regulated. Parathyroid hormone (PTH) stimulates while fibroblast growth factor-23 (FGF-23) and 1,25-dihydroxy vitamin D inhibit 1-α hydroxylation. Catabolism steps include 24-hydroxylation of 25-hydroxy vitamin D and 1,25-dihydroxy vitamin D to 24,25-hydroxy vitamin D and 1,24,25-hydroxy vitamin D, respectively, which are metabolized to calcitroic acid and then excreted by the kidney.

Vitamin D is a fat-soluble vitamin as well as a steroid hormone precursor. There are two major forms of vitamin D: ergocalciferol (D_2_) and cholecalciferol (D_3_). About 80% of D_3_ is produced by ultraviolet B (UVB) irradiation of the 7-dehydrocholesterol in the skin of the most vertebrates. Vitamin D_2_ is produced by UVB irradiation in plants and fungi (*9*). Regardless of the origin, D_2_ and D_3_ are biologically inactive forms before two hydroxylation processes: 1) 25-hydroxylation in the liver where they are converted to 25-hydroxy vitamin D (25(OH)D); and 2) 1-α hydroxylation in the kidney where D_2_ and D_3_ are finally converted to their active form 1,25-dihydroxy vitamin D (1,25(OH)_2_D) (*9*, *10*) (Figure 1). Even though the conversion of vitamin D to its active form occurs primarily in the kidneys, it can also occur in the skin, prostate, brain, pancreas, adipose tissue, skeletal muscle, heart, colon, monocyte/macrophages and neoplastic tissues (*11*). The first step in the catabolism of active vitamin D metabolites is the 24-hydroxylation process (*11*). Metabolism of vitamin D is regulated by parathyroid hormone (PTH) and fibroblast growth factor 23 (FGF23) from bone osteocytes, and induces phosphaturia in the kidney *via* sodium phosphate (NaPi-2a/c) transporters and also suppresses 1-α-hydroxylation process, while PTH also induces phosphaturia *via* PTH receptor, but in contrast to FGF23, induces 1α–hydroxylase activity (Figure 1). 1,25(OH)_2_D is responsible for the regulation of calcium and phosphate absorption from the intestine and their secretion in the kidneys while also supporting bone mineralization (*12*).

Vitamin D and its metabolites are transported in the circulation bound to proteins: 85% to vitamin D binding protein (VDBP) and 15% to albumin (*11*). When complex reaches its target cells, vitamin D dissociates from the VDBP (or albumin), enters the cells and interacts with a nuclear vitamin D receptor (VDRn). Nuclear vitamin D receptor is detected in various tissues and cells, and functions as a transcriptional factor. Liganded VDRn binds to a Retinoid-X-receptor (RXR). This heterodimeric complex can activate or suppress gene expression through binding to the elements in the promoter region of the regulated gene VDREs (vitamin D response element) initiating a formation of an assembly of nuclear transcription factors (*13*).

However, some effects of vitamin D in target cells are too rapid to be explained by stimulation of gene expression. It is recognized that 1,25(OH)_2_D also acts trough non-genomic actions that are mainly manifested as the activation of intracellular signaling pathways and consequently transcription factors that bind to VDREs (*14*). Another non-genomic action of 1,25(OH)_2_D includes the regulation of VDR binding to target receptors such as STAT1 (signal transducer and activator of transcription 1) and IKKβ (inhibitor of nuclear factor kappa-B kinase subunit beta). Non-genomic effects of 1,25(OH)_2_D are mediated by membrane VDR (VDRm) (*14*).

The biological effects of vitamin D are well known. The major targets of 1,25(OH)_2_D are intestine, kidney and bones, where, together with other calciotropic hormones, maintains calcium balance (*15*). When concentration of serum calcium is low, 1,25(OH)_2_D acts *via* VDR to increase calcium absorption from the intestine. If increased intestinal absorption is not sufficient to provide normal serum concentration of calcium, 1,25(OH)_2_D and PTH, *via* receptors release calcium from the bone and increase reabsorption of calcium from the distal tubule of the kidney (*15*).

Since the VDR expression was detected in numerous tissues it became clear that vitamin D action in many cellular targets was unrelated to mineral regulation, suggesting novel vitamin D functions (*16*). Vitamin D novel actions that are in the focus of numerous investigations include a role in cell proliferation and differentiation, regulation of the innate and adaptive immune systems, preventive effects on neurodegenerative and cardiovascular diseases, and antiaging effects (*17*).

## Effect of preanalytical factors on vitamin D concentration

Preanalytical factors can significantly influence vitamin D concentration and present a great source of variability. The mostly known and investigated preanalytical factors are endogenous and exogenous interferences. Based on the manufacturers’ declarations, generally, vitamin D is not highly sensitive to endogenous interferences of haemolysis, lipemia and icterus. However, significant differences are observed between manufacturers (Table 1).

**Table 1:** Manufacturers’ declarations on effect of haemolysis, lipemia and icterus on vitamin D measurement

**Analyzer type (manufacturer)** **Method**	**Architect i2000 (Abbott)** **CMIA**	**UniCel DxI** **(Beckman Coulter)** **Reagent Diazyme** **Immunoturbidimetry**	**Liaison (DiaSorin)** **CLIA**	**CL-1000i (Mindray)** **CLIA**	**Elecsys (Roche)** **ECLIA**	**ADVIA Centaur (Siemens)** **CLIA**	**HPLC,** **LC-MS/MS (various manufacturers)**
Haemolysis (g/L free Hb)	5	6	2	5	2	1.25	5
Icterus (µmol/L bilirubin)	513	684	684	342	1128.6	800	500
Lipemia (mmol/L of triglycerides)	5.65	11.3	6.7	17	3.39	2.8	17
Cut-off values of free Hb, bilirubin and triglycerides above which there is significant influence of haemolysis, icterus and lipemia are presented for most commonly used manufacturers of immunochemistry methods. Hb - haemoglobin. CMIA - chemiluminescent microparticle immunoassay. CLIA - chemiluminescence immunoassay. ECLIA - electro-chemiluminescence immunoassay. HPLC - high-performance liquid chromatography. LC-MS/MS - liquid chromatography - tandem mass spectrometry.							

Exogenous interferences can affect the measurement of vitamin D due to the same mechanism of metabolism (cytochrome p450 for drugs) or method used (competitive or non-competitive method with streptavidin-biotin interactions for biotin). Interferences of biotin could therefore result with the concentration of vitamin D that is over or underestimated for almost 50% (*18*).

Both serum and plasma can be used interchangeably for the measurement of vitamin D concentration (*19*). Moreover, published studies proved that vitamin D metabolite 25(OH)D is a very stable analyte in almost all storage conditions. The concentration of 25(OH)D is stable for days at room temperature, for years at -20 °C, and even up to 4 multiple freeze-thaw cycles do not change results (*20*).

However, due to the characteristic pathway and metabolism of vitamin D, some specific preanalytical issues are known. Food rich in specific ingredients like fatty fish, sea food, mushrooms and fortified milk could result in increase of vitamin D concentration. Vitamin D concentration is associated with body mass index (BMI); increase of BMI for 5 kg/m^2^ decreases vitamin D concentration for 5 nmol/L. Furthermore, women are more deficient in vitamin D than men (*21*, *22*).

**Figure 2:**
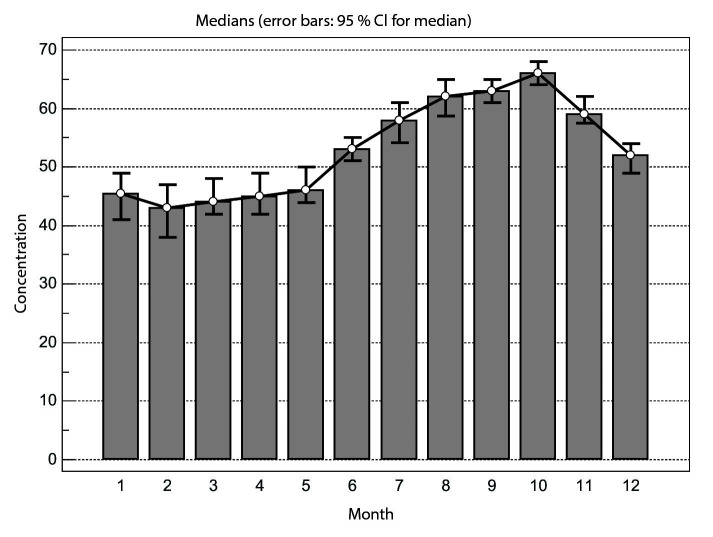
Seasonal differences in vitamin D concentration. Distribution of vitamin D concentrations measured in the outpatient unit of the Department of Clinical Chemistry, Sestre milosrdnice University Hospital Center, Zagreb, Croatia in 2018 (N = 5053). Bars are presenting median values with corresponding 95% confidence intervals. Unpublished data. CI - confidence interval.

Since almost 90% of vitamin D synthesis is influenced by the sun, skin colour and type are also important preanalytical factors. According to the Fitzpatrick scale, white and pale skin types (I to IV) could produce more vitamin D in less time (10-15 minutes exposure to sun in summer), while darker skin types (V and VI) need more time of the sun exposure (*23*). Seasonal and geographical variations affect vitamin D concentration with the highest concentrations measured at latitude 40. Vitamin D concentration is lowest in winter months, and highest in autumn months after prolonged sun exposure during the summer (Figure 2).

Also, one should be aware that immigration of the population could affect the interpretation of results (*24*, *25*). Furthermore, type of clothing coverage influences vitamin D synthesis with a significantly lower concentration in covered females (*26*). Cream with sun protection factor (SPF) above 8 blocks the synthesis of vitamin D so it is not recommended to protect the entire body surface, especially not with the highest SPF (50+) (*27*).

It is very important to be aware of all of the mentioned, and some others, preanalytical factors that could affect the interpretation of results and lead to possible misdiagnosis of vitamin D deficiency.

## Analytical challenges in determining vitamin D

The general acceptance of serum total 25(OH)D (both forms, 25(OH)D_2_ and 25(OH)D_3_) as the best biomarker for evaluating individual’s vitamin D status and assessing body’s vitamin D reserves has resulted in the development of several specific and sensitive commercial assays over the past 20 years (*28*, *29*). Its hydrophobicity, low circulating concentrations, ability to bind to lipids, albumins and VDBP, presence of multiple vitamin D metabolites and variable ratios of 25(OH)D_2_ and 25(OH)D_3_ in the circulation have defined 25(OH)D as a “difficult analyte” (*30*-*32*). Precise and accurate measurement of 25(OH)D concentration is challenging, since large differences are observed between methods. Variations between methods can be explained by: differences in vitamin D extraction, deproteinization and purification, cross-reactivity of antibodies with epimers, and/or metabolites and presence of matrix interferences (*33*, *34*). Importantly, problems in accuracy will generally lead to systematic errors and thus may cause variability of results between different measurement techniques.

Methodologies for 25(OH)D determination include high performance liquid chromatography (HPLC) with ultraviolet (UV) detection, liquid chromatography-mass spectrometry (LC-MS) or tandem mass spectrometry (LC-MS/MS) and immunochemistry, such as enzyme immunoassays (EIA, ELISA), chemiluminescent immunoassays (CLIA, ECLIA, CMIA), radioimmunoassay (RIA) or competitive protein-binding assays (CPB). Even though some analytical issues are present in all methods listed above, the current gold standard for 25(OH)D testing is isotope-dilution LC-MS/MS (*35*-*37*).

Despite this, automated immunoassays are responsible for 90% of routine 25(OH)D testing because of their high throughput, automated sample handling, and minimal manual work (*38*). Generally, techniques that are based on chromatography are more accurate than immunochemistry assays. Regarding the methods mentioned above, the separation of interfering and co-eluting C3-epimers and isobars (*e.g.* 7-α-hydroxy-4-cholesten-3-one, an endogenous bile acid precursor and 1α-hydroxy vitamin D3, an exogenous pharmaceutical compound) from the target analyte is essential, because they can overlap chromatographically with 25(OH)D_3_ or internal standard peaks and exhibit identical mass spectra, thus resulting with the positive bias when 25(OH)D is measured (*39*). This problem can be resolved by chiral phase high resolution chromatographic separation (*40*). Cross-reactivity with vitamin D metabolites is the main reason for large differences among different immunoassays, as well as between immunoassays and chromatography-based methods (*31*, *41*). Immunoassays do not detect 3-epi-25(OH)D_3._ However, small antigenic molecules like 25(OH)D are challenging target for the production of specific antibodies which commonly react with 24,25(OH)_2_D_3_ and other vitamin D metabolites (*42*). All immunoassays should measure D_2_ and D_3_ metabolites equally (with equimolar reactivity), but detection of 25(OH)D_2_ and 25(OH)D_3_ predominantly depends on the antibody specificity. Immunoassays that are able to detect 25(OH)D_2_ cannot differentiate 25(OH)D_2_ from 25(OH)D_3_ (*43*). Strong binding between the lipophilic 25(OH)D and VDBP creates competition with the capturing antibody in homogenous 1-step assays where the complete separation between 25(OH)D and VDBP is not achieved (*44*). This issue can be overcome by manual extraction to the detriment of precision. Furthermore, almost all automated immunoassays have narrow dynamic ranges and are sensitive to matrix interferences such as heterophilic antibodies. Results of immunoassays frequently either under- or overestimate 25(OH)D concentrations at the limits of the measurement range, which are often concentration ranges most important for the clinical decision (*45*, *46*). Additionally, the bias between methods is magnified by differences in the standardization of each 25(OH)D assay (*47*). As the number of 25(OH)D methods increases, standardization and harmonization of all available methods is essential. Hopefully, these goals are going to be achieved, to some degree at least, through the Vitamin D Standardization Program (VDSP) (*48*).

## Vitamin D in extravascular body fluids

Serum and plasma are considered as standard samples in a clinical laboratory, and they account for the majority of all the laboratory samples. However, some laboratory tests are measured in extravascular body fluids for diagnostic purposes. Vitamin D or its metabolites have been measured in urine, cerebrospinal fluid, pleural, peritoneal and synovial fluid, saliva and human breast milk.

Low serum vitamin D concentration was observed in patients with chronic kidney disease and dialysis patients, possibly due to VDBP loss through kidneys, and the role of the kidneys in vitamin D metabolism (*49*). Urine has become a fluid of interest due to the tubular reabsorption of VDBP and excretion of vitamin D metabolites (*50*).

Poor vitamin D status has been associated with higher risk and poor outcome in patients with multiple sclerosis (*51*). Also, the neuroprotective action of vitamin D has been observed in Alzheimer’s disease. Studies addressing vitamin D in cerebrospinal fluid are based on findings that vitamin D receptors are abundantly expressed on brain tissue, the ability of 1,25(OH)_2_D synthesis in the brain, and immunomodulatory role of vitamin D (*52*).

Vitamin D concentration is investigated in the pleural and peritoneal fluid due to its immunomodulatory effect. Higher vitamin D concentrations were observed in exudate effusions which supports a theory that vitamin D is moving toward the effusion due to an inflammatory process. Lower serum - ascites vitamin D gradient was observed in patients with spontaneous bacterial peritonitis (SBP) supporting vitamin D role in peritoneal fluid and immunological process (*53*, *54*).

In synovial fluid, 1,25(OH)_2_ vitamin D has been actively synthesized and catabolized by synovial fibroblasts which were the basis for assessment of the association between vitamin D in synovial fluid and serum in patients with rheumatoid arthritis (*55*, *56*). Saliva is interesting as a sample due to its availability and non-invasive collection, which is appropriate in the pediatric population (*57*). The nutritional value of human breast milk regarding vitamin D concentration was assessed because of previous findings that human breast milk does not fulfill the daily need for vitamin D in infants. A higher concentration of vitamin D was measured in hindmilk compared to foremilk (*58*).

Considering multiple functions of vitamin D in the immune system, its hormonal activity and regulation of calcium homeostasis, there is no doubt that investigating vitamin D in extravascular body fluids could be of diagnostic importance. However, there are some considerations that should be taken into account when interpreting the results of these studies. First of all, the sample sizes of those studies are small, which doesn’t surprise considering the fact that these are rare samples, and sometimes difficult to collect (cerebrospinal fluid, for example). Second, results should be interpreted considering study design, mostly case-control which does not provide strong evidence according to the hierarchy of scientific research (*59*). And third, the different methodology provides different possibilities regarding vitamin D concentration measurement. For instance, immunochemical tests for routine diagnostic measure 25(OH)D, which doesn’t provide information on vitamin D isoforms. LC-MS/MS and its variants enable distinction between 25(OH)D_2_ and 25(OH)D_3_, and conjugation positions of glucuronidated vitamin D. Understanding limitations of extravascular body fluids as a sample and characteristic of the method are the basis for the future study design of vitamin D in extravascular body fluids.

## Vitamin D and fertility

The main role of vitamin D is calcium homeostasis and bone mineralization. Since numerous human cells and tissues express VDR and the enzymes involved in its metabolism, vitamin D deficit is linked to the large number of diseases such as diabetes mellitus, cancer, autoimmune, infectious and cardiovascular diseases (*2*, *60*). Among others, VDR is expressed in ovarian tissue, uterus and placenta as well (*61*-*63*).

Although results are still inconclusive, existing data suggest a possible beneficial role of vitamin D in fertility. Vitamin D deficit is observed in women of reproductive age with rather high frequency, ranging from 20 to 52%, as reported by some authors (*64*). Besides that, many studies imply that vitamin D may as well have an important role in pregnancy outcomes.

Vitamin D enzymes and receptors are detected in the endometrium and have an important role in pregnancy implantation (*65*). There is evidence that the deficit of vitamin D can lead to poor placentation, which can result with hypertension and foetal growth restriction (*66*). Initial embryo implantation is regulated by vitamin D and improper implantation caused by vitamin D deﬁciency, is the cause of poor placentation, as hypothesized by some authors (*67*, *68*). Other adverse pregnancy outcomes, including preterm birth and gestational diabetes, have been observed in women with vitamin D deficiency. Additionally, vitamin D deficiency is thought to have several adverse effects on human fertility. According to the published findings, vitamin D concentration correlates well with ovarian reserve markers, especially anti-Mullerian hormone (AMH), suggesting that lower ovarian reserve in late reproductive-aged may be linked to vitamin D deficit (*69*).

Furthermore, vitamin D might have a beneficial effect on metabolic and hormonal parameters of polycystic ovary syndrome and endometriosis, which are one of the most common causes of female infertility. Although the exact mechanism of how vitamin D affects polycystic ovaries and endometriosis is not yet known, several explanations have been proposed.

*In vitro* fertilization (IVF) outcome and higher concentrations of vitamin D have been positively linked in most of the published studies. Based on the recently published data, vitamin D deficiency affects pregnancy success in women undergoing day 5 single embryo transfer (SET). Vitamin D deficiency negatively effects endometrial receptivity, which was identified as the main cause of the lower clinical pregnancy rates (*70*-*72*).

Interestingly, epidemiological studies have shown variations in human reproductive capacity throughout the year, which could be explained, at least in part, by seasonal changes of vitamin D concentrations (*73*).

In male infertility, both low and high concentrations of vitamin D in serum negatively affect spermatozoa number, their progressive movement and morphology (*74*).

Although it is not yet established whether vitamin D truly has an essential role in human fertility, the topic is worth exploring.

## Diabetes mellitus and vitamin D

Renewed interest in vitamin D, the so-called “sunshine vitamin”, has occurred recently because it has been linked to everything from cancer and heart disease to diabetes. However, most of the research is based on observational, epidemiological studies, which are important for generating hypotheses but do not prove causality. Because the destruction of β-cells usually begins in infancy or early childhood and continues until type 1 diabetes is diagnosed, it is intriguing in terms of the utility of vitamin D in people with type 1 diabetes. Currently, evidence supports that maintaining adequate vitamin D status during pregnancy, nursing, infancy, and childhood may help prevent type 1 diabetes. However, it is still unknown whether the genetics of type 1 diabetes place individuals at risk for vitamin D deficiency or whether vitamin D deficiency places individuals at risk for type 1 diabetes. Some studies have suggested that low vitamin D concentrations might increase the odds of developing type 2 diabetes and that boosting vitamin D concentrations could prevent disease onset. Newly published studies showed no likely connection between diabetes and vitamin D concentrations. On the other hand, findings from other studies suggest that high-dose supplementation of vitamin D can improve glucose metabolism to help prevent the development and progression of diabetes.

Lower incidence of type 1, and maybe even type 2 diabetes mellitus, as well as better metabolic control may be achieved by appropriate vitamin D supplementation. However, the exact mechanisms and the level of protection are not clear and need further investigation.

## Vitamin D in lung diseases

In response to proinflammatory stimuli, immune cells (*i.e.* monocytes/macrophages, dendritic cells, lymphocytes) are able to locally activate vitamin D. The generated 1,25(OH)_2_D binds to VDR, which is ubiquitously expressed in all immune cells, and regulates the transcription of various genes associated with both activation of the innate immune system and inhibition of the acquired immune response (*17*, *75*, *76*). The discovery of vitamin D immunomodulatory action together with the high prevalence of vitamin D deficiency found in patients with lung diseases of inflammatory pathogenesis (*e.g.* chronic obstructive pulmonary disease (COPD), asthma, tuberculosis (TB), respiratory tract infections (RTI) *etc.*), led to enhanced interest in investigating the potential therapeutic role of vitamin D in such conditions (*77*, *78*).

Observational studies revealed that vitamin D deficiency found in COPD patients positively correlates with disease severity, is strongly associated with the pulmonary function (*i.e.* its faster deterioration) and underlying osteoporosis, and increases the risk of RTI and exacerbations (*79*-*81*). Although *in vitro* and animal studies suggest a potential role of vitamin D in the pathogenesis of COPD, the therapeutic effects of vitamin D supplementation in COPD patients remain inconclusive (*76*, *77*, *82*, *83*).

Vitamin D deficiency found in asthma patients impairs lung function, promotes airway hyper-responsiveness and inflammation, decreases response to glucocorticoids and increases the risk of exacerbations (*83*-*85*). In contrast to *in vitro* and *in vivo* studies, which support a beneficial effect of vitamin D supplementation, results from clinical trials are inconclusive. Thus, it remains unclear whether vitamin D supplementation offers a valid treatment option in asthma patients (*77*, *85*, *86*).

The benefit of vitamin D (*i.e.* from sun exposure and cod liver oil consumption) for TB treatment has been known before the antibiotic era. The anti-microbial activity of vitamin D on *Mycobacterium tuberculosis* was later experimentally confirmed (*77*, *78*, *87*). Epidemiological studies associated vitamin D deficiency with a higher risk of active TB, increased susceptibility to TB and disease progression (*87*). While a number of studies demonstrated clinical improvement in TB patients taking vitamin D supplementation with standard TB treatment, others could not support these findings. Currently there is not enough data to define a role of vitamin D in the prevention and/or treatment of TB (*77*, *87*, *88*). Furthermore, in contrast to the discovered vitamin D association with increased risk of RTI in epidemiological studies, there are just a few clinical trials evaluating the effects of vitamin D supplementation on RTI prevention and/or treatment, with inconclusive results (*77*, *79*, *84*, *87*).

It is still uncertain if vitamin D deficiency has a role in the pathogenesis of lung diseases or it is merely a manifestation of the underlying disease (*79*). Adequate placebo-controlled interventional studies are needed to elucidate the potential causal relationship as well as the therapeutic effects of vitamin D supplementation in lung disease. Accordingly, vitamin D supplementation is not recommended above and beyond what is required for osteoporosis and fall prevention (*89*, *90*).

## Vitamin D receptor polymorphisms in rheumatic diseases

Vitamin D receptor is responsible for the biological actions of 1,25(OH)_2_D. It is a steroid, intracellular receptor which consists of 427 aminoacids located on chromosome 12. The VDR gene belongs to the family of trans-acting transcriptional regulatory factors (*91*). It encodes the nuclear hormone receptor for vitamin D and plays an important role in regulating cell differentiation and proliferation. Immunoregulatory properties of vitamin D in the cells of the immune system are mediated by VDR. The main effect of vitamin D in the immune system is the downregulation of the Th1-driven autoimmunity and suppression of proinflammatory cytokines, such as tumour necrosis factor α (TNF-α). Low vitamin D concentrations may, among other factors, be associated with VDR polymorphisms, introducing the potential immunosuppressive role of vitamin D in rheumatic diseases (*92*, *93*).

Rheumatic diseases are characterized by inflammation affecting the connecting and/or supporting structures of the body, commonly the joints, but also sometimes the tendons, bones, muscles, ligaments or even organs, and can cause loss of function in those body parts. The most common rheumatic disorder is rheumatic arthritis, which on itself covers more than 100 different disorders.

The association of over 63 polymorphisms on the *VDR* gene and disease development in rheumatic diseases have been examined. Among these, rs2228570, rs1544410, rs7975232, and rs731236 were the most common (*93*, *94*). The same polymorphisms have found to be associated with other lumbar spine pathologies (*95*). Expression of mRNA can be affected by changes in the 5'-promoter of the VDR gene, while sequence variations in the 3' untranslated region (UTR) can alter mRNA stability and protein translation efficiency. However, the changes can take place in exons also, consequently leading to changes in the protein sequence.

Methods used for polymorphism detection include: restriction fragment length polymorphisms (RFLP), DNA sequencing techniques and real time PCR with hydrolysing or hybridizing probes.

However, heterogeneity in results was found by various authors (*91*). Polymorphisms of VDR may be the key to understand this heterogeneity. In understanding the VDR gene polymorphism as a significant risk factor for rheumatic diseases, several facts need to be considered. Source of heterogeneity among studies might have been due to other factors, such as diversity in the population (age, ethnicity, sun exposure and dietary vitamin D intake, *etc.*), study design and genotyping methods. Some studies have shown that the ethnic (genetic) background, gene-gene or gene-environment interactions and lifestyle (sun exposure, dietary vitamin D intake and obesity) might have a significant impact on increased risk of rheumatic diseases in association with polymorphisms (*96*).

In conclusion, nowadays vitamin D seems to be an inevitable laboratory test in diagnosis, management and treatment of various diseases, since; undoubtedly, many studies have found a significant association of vitamin D concentration and disease development and progression. However, many of these promising original studies were not confirmed on randomized-controlled trials that have investigated the efficiency of vitamin D supplementation. Furthermore, high sensitivity to many preanalytical factors coupled with method heterogeneity and lack of harmonization point to caution in interpretation of laboratory results. If the increased financial burden of vitamin D determination and supplementation will result in a positive impact on patients’ health, remains to be seen.
